# Manganese mono-boride, an inexpensive room temperature ferromagnetic hard material

**DOI:** 10.1038/srep43759

**Published:** 2017-03-06

**Authors:** Shuailing Ma, Kuo Bao, Qiang Tao, Pinwen Zhu, Teng Ma, Bo Liu, Yazhou Liu, Tian Cui

**Affiliations:** 1 State Key Laboratory of Superhard Materials, College of Physics, Jilin University, Changchun, 130012, China

## Abstract

We synthesized orthorhombic FeB-type MnB (space group: Pnma) with high pressure and high temperature method. MnB is a promising soft magnetic material, which is ferromagnetic with Curie temperature as high as 546.3 K, and high magnetization value up to 155.5 emu/g, and comparatively low coercive field. The strong room temperature ferromagnetic properties stem from the positive exchange-correlation between manganese atoms and the large number of unpaired Mn 3d electrons. The asymptotic Vickers hardness (AVH) is 15.7 GPa which is far higher than that of traditional ferromagnetic materials. The high hardness is ascribed to the zigzag boron chains running through manganese lattice, as unraveled by X-ray photoelectron spectroscopy result and first principle calculations. This exploration opens a new class of materials with the integration of superior mechanical properties, lower cost, electrical conductivity, and fantastic soft magnetic properties which will be significant for scientific research and industrial application as advanced structural and functional materials.

Ever-growing demanded advanced magnetic materials for application in designing power electronic components, magnetic recording, magnetic refrigeration as well as spin-electronic have recently been investigated both from scientific and technological aspects^1^,^2^,^3^,^4^. Nowadays, the rapid development of micro-electromechanical systems (MEMS) devices, magnetic read, magnetic sensor, high speed motors, magnetic springs have given fresh impetus to reinforce mechanical property of ferromagnetic materials, such as high hardness, low compressible ability and ductile ability^5^,^6^,^7^,^8^,^9^. However, most of ferromagnetic materials (FM) are metal alloys, magnetic ion oxides, rare-earth transition metal (R-T) intermetallic compounds which are dominated by the metallic bonds or ionic bonds, a bond feature that is unfavorable to resist shear strain and compress. The relatively low hardness, typically below the value of 5 GPa, of traditional magnetic materials inevitably impede the application of traditional magnetic materials in harsh conditions^9^,^10^,^11^,^12^,^13^,^14^. To strengthen the mechanical properties of ferromagnetic materials, fabricating composite, ageing process, doping and controlling microstructure have been attempted to enhance mechanical properties^7^,^8^,^9^,^15^,^16^,^17^. However, the mechanical properties of ferromagnetic materials still fall far short the requirements of industrial applications^18^. Furthermore, for massive and industrial scale production, the earth abundant resources are most attractive due to high cost of rare-earth metals. Therefore, low cost mono-phase materials, combined the high temperature ferromagnetic with fantastic mechanical properties, are attractive for multifunctional application^19^.

Due to the strong covalent bonds between metalloid atoms and bonds between transition metal (TM) atoms and metalloid atoms, transition metal metalloid compounds typically present outstanding mechanical properties^20^,^21^,^22^,^23^. Manganese borides were one of the most promising transition metal (TM) borides because of their high hardness, low compressibility, high melting point, high electric conducting, wear resistance, thermal conduction and even room temperature magnetic properties^24^,^25^,^26^,^27^. Furthermore, from a more technological point of view, it is noteworthy that their INVAR behavior and high glass forming ability (GFA) to form bulk glass materials (BGMs) may be more useful in industrial applications^6^,^28^,^29^. However, lower boron contents generally contribute to relatively soft mechanical properties and the higher boron content manganese borides basically exhibit weak paramagnetic behavior. Consequently, the proper boron content and suitable crystal structure may facilitate ferromagnetic properties and outstanding mechanical property. Synthesis of orthorhombic MnB was first reported by Kiessing in 1950^30^. Due to the proper boron contents, MnB was reported to have high Curie temperature, Tc, among transition metal borides (TMBs). Although FeB-type MnB possess outstanding ferromagnetic property, the magnetic parameters as well as the origin of strong ferromagnetic are still lacking in the literature^31^. This is probably because the magnetic exchange correlation between manganese atoms is complex and the intricate nature of chemical bonds. Thus, to instruct future material design, it is necessary to explore the relationship between the electron configuration and its physical property.

In this letter, to seek superior mechanical ferromagnetic materials, polycrystalline manganese mono-boride has been successfully synthesized at high pressure and high temperature (HPHT). MnB exhibits high Curie temperature, high saturated magnetization and low coercive field. Furthermore, the mechanical property, Vickers hardness (Hv), is enhanced by introducing strong covalent boron bonds. The combined mechanical, room temperature ferromagnetic, electrical conductivity and chemical inertness make this material promising candidates for engineering and technological applications.

## Experiments

Pure manganese powder (99.95% in purity) and amorphous boron powder (99.5% in purity) with a ratio of 1:1 was ground together with an agate mortar and pestle until a uniform raw mixture achieved. The raw mixture was pressed into a pellet with 4 mm in diameter and 2.5 mm in height by means of hydraulic pressing. Hexagonal BN capsule was used as an insulate material to prevent the raw material reacting with carbon heater placed in the pyrophyllite cube. The synthesis experiments were carried out on SPD6 × 600 T cubic anvil apparatus with target pressure 5.0 GPa, temperature 1600 K to 2200 K and duration time of 15 min to 60 min. Finally the sample temperature was rapidly quenched to room temperature by switching off power supply and then decompressed to ambient pressure (AP). Powder X-ray diffraction (XRD) of the as-synthesized powder was performed with angle-dispersive mode at the beamline 16-BMD of the Advanced Photon Source (APS) Argone National Laboratory. GSAS program suite was used to perform Rietveld refinement^32^. The microstructure and the chemical compositions was observed by the scanning electron microscopy (SEM, JSM-6480LV) and the chemical composition were detected by the energy dispersive spectroscopy (EDS). The density of the specimens was measured by Archimedes methods with MDY-2 densimeter at room temperature (RT). The electrical resistivity was investigated with Van Der Pauw method. Temperature dependent dc magnetization susceptibility (χ_dc_) curves of polycrystalline MnB from 5 K to 800 K were measured by Quantum Design MPMS 3 (superconducting quantum interference device, SQUID) with magnetic field of 500 Oe. To investigate the magnetic structure transition temperature of manganese mono-boride, differential scanning calorimetry (DSC) measurements from room temperature (RT) to 800 K were conducted utilizing a Rigaku TG-DTA 8120 system. Powder samples were heated up to 800 K at a rate of 5 K/min and soaked at the temperature of 800 K for 5 min under argon atmosphere. Then, the temperature was air cooled to the room temperature at the rate of 5 K/min. X-ray photoelectron spectrum (XPS) measurements were conducted with an X-ray source equipped with an Mg anode in an ultra-high vacuum chamber at a base pressure of 10^−10 ^ mbar.

First principles calculations were conducted with Vienna ab initio simulation package (VASP), based on density functional theory (DFT) using projector augmented waves (PAW) method^33^. The Perdew-Burke-Ernzerhof (PBE) version of generalized gradient approximation (GGA) was used for determining the exchange and correlation energy^34^. For more reliable convergent test, a cutoff energy of 500 eV and Monkhorst-Pack k mesh of 0.03 × 2 πÅ^−1^ were utilized to ensure that the total energies are well converged to better than 1 meV/formula units (f.u.). The Mulliken populations were obtained by density functional plane wave technique implemented in the CASTEP code and the ultrasoft Vanderbilt pseudopotentials (USPP) with PBE-GGA was chosen^33^,^35^,^36^.

## Results and Discussions

**Figure 1:**
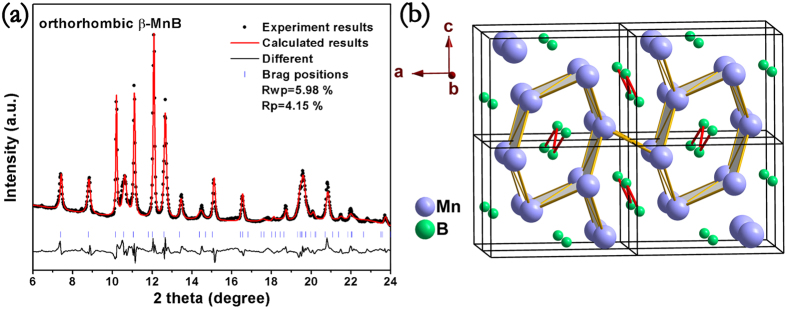
(**a**) Rietveld refinement pattern of the FeB-type MnB. Solid dots: observed dots, red line: calculated curve, green vertical line: Bragg positions, blue line: the different curve between the observed and the calculated curve. (**b**) The crystal structure of the FeB-type MnB. The purple spheres represent the Mn atoms and the green spheres represent B atoms.

Figure 1(a) displays the X-ray diffraction (XRD) pattern of the pure product synthesized at 5.0 GPa, 1600 K with a holding time of 60 min. X-ray diffraction pattern denotes that the as-synthesized specimen is highly crystalline and no lines of impurities were detected. Rietveld refinements discover that MnB adopts an orthorhombic Pnma (no. 62) structure, isotropic with FeB with lattice parameters a = 5.6377 Å, b = 2.9945 Å, c = 4.1795 Å. The refined crystal structure of FeB-type MnB is compiled in the Table 1. Rietveld refinements results and the later EDS measurements reveal that the obtained specimen does contain some manganese defects. Figure 1(b) shows the crystal structure of FeB-type MnB. There is one B and one Mn atom in nonequivalent atomic Wyckoff sites of Mn (4c) and B (4c). The boron atoms have the [BMn6B2] coordination polyhedral. It is worthy to be noted that the inter-atomic distances of B-B bonds in the zigzag chains is 1.7966 Å with bonds angle of 111.9° forming zigzag chains (ZC) and six manganese atoms at different distance form triangular prism. The zigzag B-B bonds length is slightly larger than the sum of covalent atomic radius, 1.68 Å, while the Mn-B bonds is 2.18 Å far shorter than the sum of atomic radius of manganese and boron (2.45 Å)^37^.

**Figure 2:**
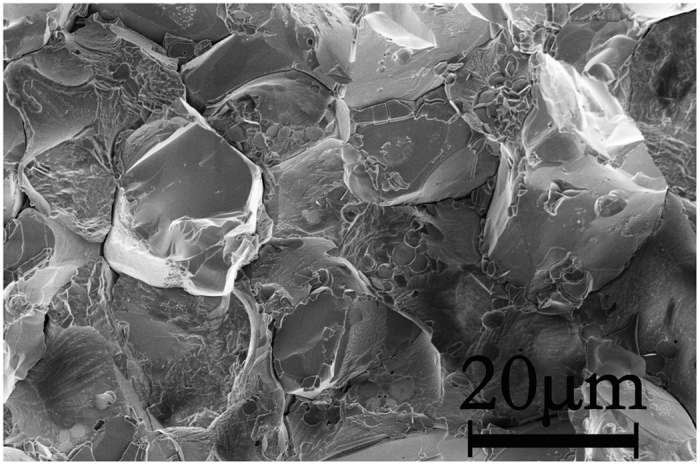
Scanning electron microscopy (SEM) image of the as-obtained pure MnB bulk specimens.

To investigate the morphology of synthesized specimen, the fracture surface of specimen was observed by scanning electron microscope (SEM). As can be seen in the SEM examinations, Fig. 2, that the product synthesized by HPHT is highly crystalline exhibiting an equilibrated microstructure with homogeneous grains size of about 30 μm. EDS was utilized to verify the chemical composition and the element purity of the synthesized specimen. The EDS result reveals that the synthesized specimen is chemically non-stoichiometric Mn_0.95_B signifying that there are some manganese defects. The density of polycrystalline pellets is 6.21 g/cm^3^ which are 96.2% of the theoretical density. High relative density ensures that the measurement of hardness is the intrinsic property of polycrystalline specimen^38^. Besides, it is worthy to note that the low density of MnB, but with high hardness, can be beneficial for the applications where light material is an asset. The electrical resistivity of MnB is measured to be 1.22 × 10^−7 ^Ω m, which is comparable to most of metal alloys^39^.

**Figure 3:**
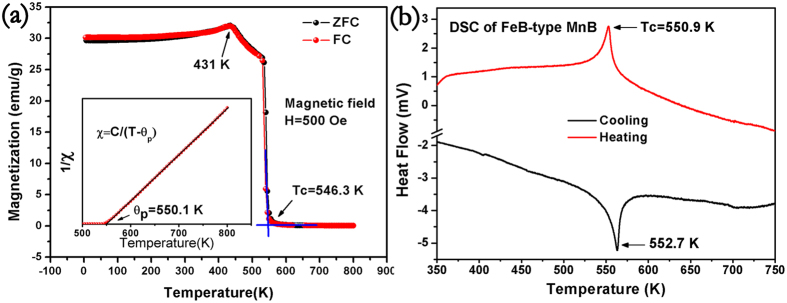
(**a**) Temperature dependence of zero field cooled (ZFC, red color) and field cooled (FC, black color) magnetizations in an applied field of 500 Oe for FeB-type MnB. The reciprocal magnetic susceptibility plotted against temperature above Curie temperature is shown in the inset. (**b**) Differential scanning calorimetry data for FeB type MnB powder synthesized by HPHT.

Successful synthesis of the well-crystalline polycrystalline MnB specimen allows us to investigate their magnetic properties. The magnetic property of the synthesized FeB-type MnB was investigated by magnetic properties measurement system (MPMS). Figure 3(a) shows the temperature dependence of magnetization in the zero field cooled (ZFC) and field cooled (FC) process with an applied magnetic field of 500 Oe from the temperature of 5 K to 800 K for polycrystalline MnB specimen. From the temperature dependent magnetization curve, it is obvious that the magnetization decreases drastically at the Curie temperature, 546.3 K. High Curie temperature manifests the presence of strong ferromagnetic (FM) interaction between manganese atoms. This Curie temperature is about 30 K lower than FeB-type MnB synthesized by arc melted method^31^. Futhermore, there is an abnormal transition of magnetic susceptibility at the temperature of 431 K and it is tentatively defined as the changing of easy magnetization axis. This abnormal transition has been confirmed to be its intrinsic property by different specimens and instruments measurements. Above the temperature of 500 K, the reciprocal susceptibility versus temperature is shown in the inset of Fig. 3(a). By fitting the reciprocal magnetic susceptibility (χ) plotted against temperature (T) with Curie-Weiss law, Eg. 1,


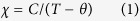


the Weiss constant, θ, can be confirmed to be 550.1 K with the Curie constant (C) of 1.15 × 10^−5^ emu·K·mol^−1^. Using equation, *μ*_eff_ = 2.83C^1/2^, and the obtained Curie constant suggests a magnetic moment near 2.71*μ*_B_.

The Curie temperature measured by Zhu *et al*. is nearly 30 K higher than our results^31^. Consequently, it is more reliable to confirm the Curie temperature by another method. Ferromagnetic (FM) structure transform to paramagnetic (PM) is usually accompanied with an exothermic or endothermic peak. To confirm Tc, the differential scanning calorimetry (DSC) data of FeB-type MnB were presented in the Fig. 3(b) for 23.77 mg FeB-type MnB powder sample. There is an obvious exothermic peak at 550.9 K in the heating procedure and an endothermic peak at 552.7 K in the cooling procedure. The temperature of exothermic and endothermic peak is consistent with the Curie temperature determined by the temperature dependent magnetization curve. According to the previous reports, Curie temperature is strongly dependent on the mean diameter of grain size and the applied magnetic field^40^. So, the Curie temperature of MnB can be safely confirmed to be 546.3 K for the HPHT synthesized specimens when the applied magnetic field is 500 Oe.

**Figure 4:**
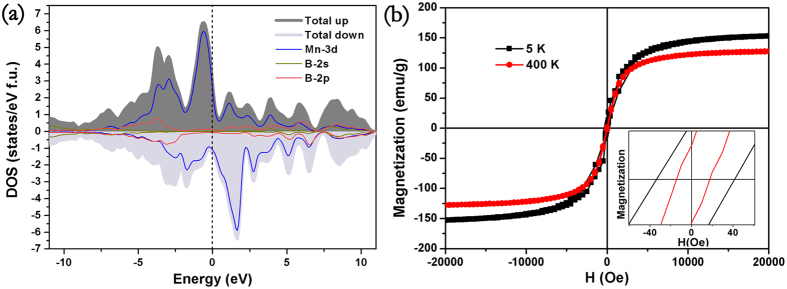
(**a**) Spin-polarized DOS of MnB. The dark gray and light gray represent spin-up and spin down states, respectively. (**b**) Hysteresis loops for MnB synthesized by high temperature and high pressure measured by MPMS at 5 K and 400 K, respectively. The inset shows the enlarged hysteresis loops at the low applied field.

Among the six manganese borides, Mn_2_B is non-magnetic, MnB_2_ and Mn_3_B_4_ exhibits anti-ferromagnetic behavior and MnB_4_ possesses paramagnetic property^24^,^25^,^26^,^27^. The absence of ferromagnetic spin order of other manganese borides is attributed to the insufficient interaction between the manganese atoms to stabilize the ferromagnetic spin order. The ferromagnetic spin order of MnB is the result of partially filled Mn valence 3d-orbital electrons, Mn-Mn exchange correlations as well as any other electronic factors^41^. Based on the Bethe-Slater curve, the exchange interaction is negative for the manganese metal^31^. In the synthesis of manganese mono-boride, boron atoms are incorporated into the matrix of manganese lattice, which expands the distances between manganese atoms. Consequently, based on the empirical Bathe-Slater curve, the negative exchange-correlation integral of antiferromagnetic α-Mn metal becomes positive. According to the Stoner model, spontaneous magnetization appears when the condition N_TM_ × I_TM_ > 1 is satisfied, where N_TM_ is the non-polarized partial density of states at Fermi energy level and I_TM_ is the exchange-correlation integral between manganese atoms^41^. The exchange correlation integral between manganese atoms is 0.41 eV calculated by Janak^42^. Besides, the density of states (DOS), shown in Fig. 4(a), is about 4.1 states/eV/f.u. which is slightly larger than the value of Park *et al*.^43^. Based on the positive exchange correlation integral and high density of states at the Fermi level, FeB-type meets the Stoner model which ensures the spontaneous magnetization.

The magnetic properties were further measured by magnetic hysteresis measurements, which were measured with the field swept from −20 KOe to 20 kOe. The hysteresis loops at 5 K and 400 K were measured and the results are shown in the Fig. 4(b). The saturated magnetization at 5 K is about 155.5 emu/g and there is hardly any hysteretic behavior. To exclude the possibility of super-paramagnetic (SPM), the hysteresis of MnB at 5 K and 400 K was fitted by Langevin equation and the results rule out the possibility of SPM^44^. The origin of the small coercive field may come from high relative density of the specimen and the large crystal grain size. The obtained saturation magnetization 155.5 emu/g at 5 K is higher than the value 124 emu/g obtained by Zhu *et al*. Because there is an obvious impurity diffraction peak at about 42 degree, higher saturated magnetization may stem from the higher sample purity than that the specimen synthesized by arc melted^31^. According to the saturated magnetization at 5 K, the manganese atoms in FeB-type MnB carries a magnetic moment 1.83 *μ*_B_/f.u. which is consistent with our DFT calculation result of 1.87*μ*_B_ and 1.91*μ*_B_ by Kervan *et al*.^27^.

Magnetic properties of other TMBs have been investigated by previous research and the experimental results are tabulated in Table 2. Besides FeB and CoB, few TMBs are reported to be room temperature (RT) ferromagnetic^45^,^46^. The most strikingly feature of MnB is higher magnetization and lower coercive field with respect to other FM transition metal borides (TMBs). Compared with other TMBs, the saturated magnetization of MnB is nearly twice of FeB and triple of CoB. Furthermore, even compared with the widely used strong soft ferromagnetic materials, such as Fe_3_O_4_, NiMnIn, and CoFeO_4_, FeB-type MnB has a higher saturated magnetization, Ms, and lower coercive field, Hc. Those outstanding magnetic features may endow MnB promising soft room temperature ferromagnetic materials. It is not surprising that MnB has a higher magnetization. The electron configuration of manganese atoms is Mn-[Ar]3d^5^4s^2^ which indicate that manganese atoms possess the largest number of unpaired spin electrons. Therefore, in the mono-borides, the Mn ions carries magnetic moment of 1.83 *μ*_B_ and Fe ions and ions Co carries smaller 1.12 *μ*_B_ and 0.28 *μ*_B_, respectively. In addition, compared with traditional ferromagnetic materials, MnB has a comparable high Curie temperature which is suitable to use in the high temperature conditions. Owing to the high GFA of TMBs to form BMGs, BMGs MnB may exhibit more superior soft ferromagnetic materials in the future^6^,^28^,^29^.

**Figure 5:**
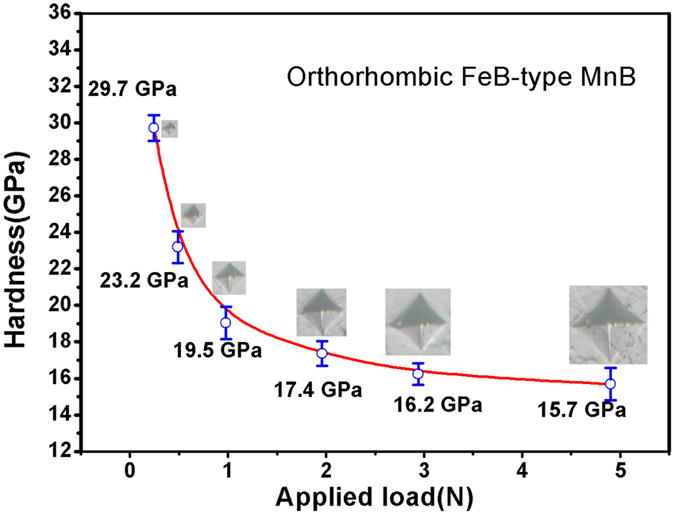
Vickers hardness of the FeB-type MnB sample as a function of the applied load ranging from 0.245 N (low load) to 4.9 N (high load). The corresponding Vickers hardness ranging from 29.7 GPa to 15.7 GPa at low load and high load, respectively. Typical optical images of the indentation made at different load are shown in the picture up the hardness data of different load.

Due to the exact nature of the bond in ferromagnetic crystals, a major challenge hindering the application of magnetic materials is the relatively soft mechanical properties and chemical instability^9^,^10^,^11^,^12^,^13^,^14^. Considering that the boron chains have been successfully incorporated into the lattice of manganese metals, the mechanical properties of manganese mono-boride may be higher than that of traditional ferromagnetic materials. So, after cutting and polishing, micro-indentation experiments were performed on the polished specimen surface at different applied load between 0.245 N and 4.9 N for MnB. Vickers micro-hardness (Hv) of MnB was calculated according to the following equation E.g. 2,


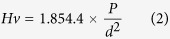


where P is the applied load (in N), d is the arithmetic mean of the two diagonals of the indentation (in micrometers) at a given applied load, Hv is the Vickers micro-hardness. The load dependent Vickers micro-hardness data for MnB are shown in the Fig. 5. It is clear that there is an inverse relationship between the applied load and Vickers micro-hardness. MnB has an asymptotic Vickers hardness of 15.7 GPa at 4.9 N and a maximum Vickers hardness of 29.6 GPa at 0.245 N. As shown in the Table 2, the AVH of MnB is far higher than traditional ferromagnetic materials. With those results, it denotes that the Vickers hardness of manganese metal has been drastically enhanced by incorporating zigzag boron chains into manganese atoms matrix. Other than benefits of ferromagnetic property, superior mechanical properties will bring it potential candidate to replace other conventional ferromagnetic materials in harsh application conditions.

**Figure 6:**
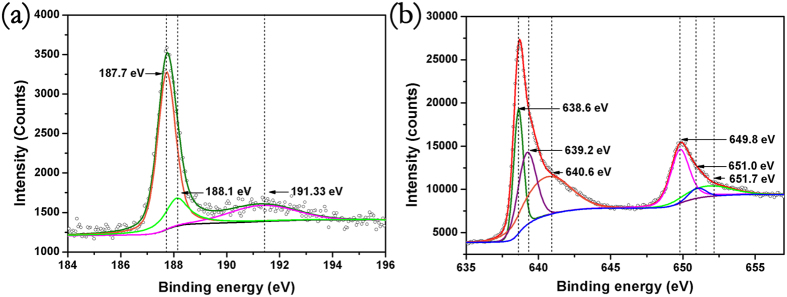
XPS spectra of MnB specimen synthesized by high pressure and high temperature which has been cleaned by the Ar + sputtering with 180 s. (**a**) B 1 s spectrum of MnB. (**b**) Mn 2p^3/2^ and 2p^1/2^ spectrum of MnB.

Generally, the hardness and magnetic property are especially sensitive to the electron configuration. Hardness prefers high bond density, high electronic density as well as strong covalent bonds framework, while the metallic bonds and ionic bonds are detrimental to hardness^47^. For the lack of convincing experimental evidences about bonds characteristics in TMBs, many incompatible models have been proposed to illuminate the physical properties^48^,^49^,^50^,^51^,^52^. To further design mechanically hard ferromagnetic materials in the future, it is necessary to investigate the chemical bonds feature and electron configurations. One of the most promising methods to explore the bond characteristic of TMBs would be X-ray photoelectron spectrum (XPS) combined with first principle calculations. Figure 6(a) shows the B-1s spectrum with the binding energy from 184.0 eV to 196.0 eV and (b) shows the Mn-2p of MnB with the binding energy from 634.0 eV to 656.0 eV. B-1s core level spectrum as shown in Fig. 6(a) has a tiny component and two major distinct components. The weak peak at 191.3 eV would be assigned to the surface defects. As to the BE at 187.7 eV, it can be assigned to the B-B bonds in zigzag boron chains, while the BE at 188.1 eV may belong to Mn-B interactions which is compatible with other TMBs^53^. The shorter bond lengths combined with marginally binding energy (BE) shift compared with boron elementary substance manifest boron bond covalent feature. De-convolution of the two distinct unsymmetrical peaks of Mn 2p suggests the presence of three chemically distinct manganese states. There is a pair of peaks located at the binding energy of 638.6 eV, 649.8 eV and another two pairs of peaks located at the binding energy (BE) of 639.2 eV, 651.0 eV and 640.6 eV, 651.7 eV. The binding energy of Mn 2p^3/2^ and Mn 2p^1/2^ in manganese metal and manganese alloy is close to 638.6 eV and 649.8 eV, such as Al_56_Mn_8_Pd_36_ and Al_70_Mn_9_Pd_21_^54^,^55^. The compatible binding energy with manganese metal and manganese alloy suggests that the bonds between manganese atoms are metallic bonds. While the pair of peaks at 639.2 eV, 651.0 eV is higher than pure manganese metals and alloys. This higher BE suggest the hybridizations between manganese 3d orbitals and boron 2p orbitals which has been demonstrated by density of states in Fig. 4(a). Another pair of small peaks at the BE of 640.6 eV, 651.7 eV can be tentatively assigned to the manganese defects in the crystal structure or caused by HPHT.

One observes in the Fig. 4(a) that Mn-3d orbitals and B-2p orbitals form some hybridization, which contributes to the covalent component of Mn-B bonds. Futhermore, the atomic average Mulliken charge are 0.62 and −0.62 for B and Mn which is compatible with XPS results. This fact denotes that there is some ionic component between Mn-B bonds. In addition, the calculated Mulliken overlap population of Mn-B bonds is 0.24 and it also suggests that there is some ionic component in Mn-B bonds. XPS experiment results suggest that B-B bond is of strong covalent character. Compared with the Mulliken population of C-C bonds in diamond (0.75) and graphite (1.03), the B-B bonds Mulliken population, 1.52, is high which imply its strong covalent feature^56^.

Considering the above electron configuration of MnB, a possible model for bond feature in MnB is given as follows: there are covalent zigzag boron chains, ionic and covalent interaction between manganese and boron atoms, metallic interaction between manganese atoms in MnB crystal. Therefore, the superior mechanical properties of ferromagnetic FeB-type MnB are mainly attributed to the zigzag boron chains running through the manganese matrix. Knowledge concerning the chemical state of elements in MnB is of great value to unravel the relation between chemical bonds characteristics and its mechanical properties. Besides, the theories to design hard materials can be utilized in the synthesizing of mechanically hard ferromagnetic materials in the future.

## Conclusions

We present a controlled HPHT method to synthesize polycrystalline MnB, which demonstrate its feasibility of synthesizing high density and well crystalline functional bulk materials. Due to the proper boron content, the exchange correlation between manganese atoms is positive which ensures the spontaneous magnetization. The synthesized specimen exhibits high saturated magnetization, high Curie temperature, low coercive field and outstanding mechanical properties. XPS results demonstrate that introducing the covalent zigzag boron chains brings superior mechanical properties. The integration of fantastic ferromagnetic properties with mechanical properties opens a new class of materials utilized in the areas where strong ferromagnetic and high mechanical strength is highly desired.

## Additional Information

**How to cite this article:** Ma, S. *et al*. Manganese mono-boride, an inexpensive room temperature ferromagnetic hard material. *Sci. Rep.* **7**, 43759; doi: 10.1038/srep43759 (2017).

**Publisher's note:** Springer Nature remains neutral with regard to jurisdictional claims in published maps and institutional affiliations.

## Figures and Tables

**Table 1:**
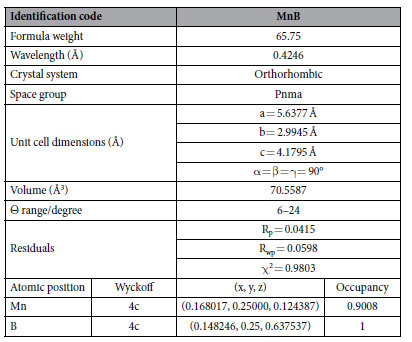
Experiment data and the crystal details obtained from the Rietveld refinement of MnB.

**Table 2:** Experiment magnetic parameters of transition metal mono-borides and other ferromagnetic materials.

Compounds	Ms (emu/g)	Tc (K)	Hc (Oe)	Hv (GPa)
MnB	155.5	546.3	15.9	15.7
FeB	80.5^45^	590^45^	10.0^45^	16.8^13^
CoB	22.4^57^	477^57^	–	–
Fe_3_O_4_	63.5^46^	858^40^	140.0^46^	2.9^10^
FeNi	52.2^9^	590^9^	63.3^9^	6.6^9^
NiMnIn	39.5^58^	325^59^	130.0^12^	7.2^12^
FeSiAl	78.0^60^	–	–	1.5^61^
CoFe_2_O_4_	56.6^62^	465^63^	1.4 × 10^4^ 62	6.3^11^
